# Topics of Public Interest

**Published:** 1914-11

**Authors:** 


### TOPICS OF PUBLIC INTEREST.

---

**The Goat Island Spring**, Niagara Falls, well known to generations of visitors as a picturesque spot of refreshment on a hot day, has been supposed to be free from contamination. Dr. E. E. Gillick, the Local Health Officer, reports that examinations have shown 1400 colonies per c.c., nine out of ten of which revealed colon bacilli. The spring has been closed.

---

**The Fly Poison Peril.** During July and August, Child Betterment, published by Dr. Frank G. Lydstrom of Chicago, collected from the daily press 35 cases of serious results in children from drinking fly poison. 5 cases were fatal. It is not thought that this is the complete list of casualties. As we have pointed out, there is danger in the fly campaign, especially when children are enlisted in it. The fly, so far as we are aware, is not intrinsically dangerous. It is not poisonous, it is not concerned specifically in the conveyance of any infection. It is called filthy but it simply reflects back upon man, his own filthiness or that which he maintains in his use of domestic animals. The Committee on Pollution and Sewerage of the Merchants' Association of N. Y. is responsible for the statement that flies cost the country 350 million dollars annually. This means $17.50 per average family of five and we do not consider it correct. Screens are somewhat expensive but they exclude other insects, including the mosquitoes which are specific carriers of malaria, mammalian vermin, a large amount of dust and thieves. They would be worth what they cost if there were no flies. What few flies enter a properly screened house can be caught in sugar-formalin solutions or on fly paper or by an occasioal "swat" at an expense of about 25 cents per year per family. If garbage, manure, cess pools and street dirt are covered and removed as they should be irrespective of the danger of the conveyance of germs by flies, flies would be reduced to a negligible factor in disease and economic waste. "The progeny of a single pair of flies, assuming that they all live, . . . at the end of the summer, would occupy a space of over 14 million cubic feet, equivalent to the Woolworth Building." Yes, and if Adam and Eve had been created at the time that Columbus discovered America, and had procreated in accordance with eugenic and moral requirements at the rate of ten children per couple, there would now be in the world more persons than would fill it beyond its capacity for raising food—and not assuming that any of the early generations had survived beyond the ordinary span of life either. As a matter of fact, the numbers of any form of animal or vegetable life are controled by a variety of factors and no mathematic series is realized in nature. Let us recognize how and why the fly is a sanitary menace, exclude it, so far as possible, from habitations, attack its pabulum which is, after all, what makes the fly dangerous, and avoid exaggeration and fads.

---

**Increase in Railroad Rates.** The Interstate Commerce Commission approved, September 30, the raise of rates in the eastern trunk line territory to 2¼ cents per mile on mileage. Without questioning the wisdom of the decision, it must be admitted that this is a further step in the degradation of the dollar and, therefore in the so-called high cost of living. Higher wages ultimately, mean higher prices.

---

**Germ of Infantile Paralysis.** At the 25th jubilee anniversary of John Hopkins Medical School, Dr. Flexner made a further report, corroborating his discovery and adding another point to the evidence of specificity: communication of the disease from cultures to monkeys. The virus, previously shown to be filtrable, contains a minute but visible bacterium, approximately 1-45 the size of the smallest previously accepted micrococci. One more ultra-microscopic, putative germ has been crossed off the list. With our past experience in so-called ultra-microscopic germs, including that of syphilis, which has proved to be one of the largest infectious microorganisms, scepticism as to the existence of forms of life very much smaller than the ordinary micrococci, naturally increases.

---

**The Batavia Hospital** will receive $2000 from the estate of the late George J. Austin.

---

**The Middlemen as Factors in the High Cost of Living.** We have received a circular with an illustration on the cover, of the raise in price of potatoes. The schedule is as follows: cost to produce 43 cents, to dealer 50c, to commission man 65c, to grocer 90c, to consumer $1.20. The theory of eliminating the middlemen is all right but, in practice it works out about as follows: Market price, delivered, 90 cents; price along state road $1.25; and out on an untraveled road, the farmer will imply that we are a mixture of Jew. Scotchman and Connecticut Yankee and reluctantly consent to dump a bushel of unwashed culls into the machine for 80 cents. And we have received half a dozen circulars about apples at prices varying from a fancy maximum for an uncertain boxful to a bushel at about $1.50 for inferior varieties, while the trees are literally breaking under the weight of fruit and pigs are gathering the crop.

**Vacational Typhoid.** Several years ago, at one of the A. M. A. meetings at Atlantic City, we expressed the opinion that the autumnal rise of typhoid incidence was due largely to the superior opportunities for infection enjoyed at summer resort and ordinary wells. To put it mildly, this opinion did not accord with the majority view. However, we have kept on insisting on this factor and have also stated that a city should be held responsible, not for its total incidence of typhoid but for that occurring in its residents and, even for them, with due regard to possibility of extra-mural infection. The Buffalo Health Department reports 91 cases of typhoid in September and adds the foot-note that about 75 per cent. of the patients had been out of town, mostly at summer resorts.

**The Buffalo Homoeopathic Hospital** has recently opened a pathological laboratory, the gift of Mrs. Joseph T. Jones.

**A Series of Lectures on Medical Aspects of Cancer** will be given by Dr. L. Duncan Bulkley at 4.15 p. m., Wednesdays, at the N. Y. Skin and Cancer Hospital, 2d Avenue and 19th street, preceded by a half-hour clinic. The schedule is as follows: Physicians will be admitted on presentation of professional card. November 4, Nature of Cancer; November 11, Frequency and geographical distribution of Cancer; November 18, Metabolism of Cancer; November 25, Relation of Diet to Cancer; December 2, Medical Treatment of Cancer; December 9, Clinical Considerations and Conclusions.

